# Marker-Less Lung Tumor Tracking from Real-Time Color X-Ray Fluoroscopic Images Using Cross-Patient Deep Learning Model

**DOI:** 10.3390/bioengineering12111197

**Published:** 2025-11-02

**Authors:** Yongxuan Yan, Fumitake Fujii, Takehiro Shiinoki

**Affiliations:** 1 Graduate School of Sciences and Technology for Innovation, Yamaguchi University, Ube 755-8611, Japan; 2 Graduate School of Medicine, Yamaguchi University, Ube 755-8505, Japan; shiinoki@yamaguchi-u.ac.jp

**Keywords:** tumor tracking, KV X-ray, IGRT, deep learning, fluoroscopic image

## Abstract

Fiducial marker implantation for tumor localization in radiotherapy is effective but invasive and carries complication risks. To address this, we propose a marker-less tumor tracking framework to explore the feasibility of a cross-patient deep learning model, aiming to eliminate the need for per-patient retraining. A novel degradation model generates realistic simulated data from digitally reconstructed radiographs (DRRs) to train a Restormer network, which transforms clinical fluoroscopic images into clean, DRR-like images. Subsequently, a DUCK-Net model, trained on DRRs, performs tumor segmentation. We conducted a feasibility study using a clinical dataset from 7 lung cancer patients, comprising 100 distinct treatment fields. The framework achieved an average processing time of 179.8 ms per image and demonstrated high accuracy: the median 3D Euclidean tumor center tracking error was 1.53 mm, with directional errors of 0.98±0.70 mm (LR), 1.09±0.74 mm (SI), and 1.34±0.94 mm (AP). These promising results validate our approach as a proof-of-concept for a cross-patient marker-less tumor tracking solution, though further large-scale validation is required to confirm broad clinical applicability.

## 1. Introduction

According to data from the World Health Organization, lung cancer has the highest incidence and mortality rates among all cancers [1]. Radiotherapy remains one of the primary treatment modalities for lung cancer [2,3,4]. Lung tumors exhibit individualized periodic displacement due to respiratory motion, which can easily lead to discrepancies between the planned and actual irradiation areas, thereby affecting the accuracy of radiation dose delivery and increasing the risk of complications. Image-guided radiation therapy (IGRT), which relies on continuous or quasi-continuous imaging during treatment to monitor the spatial positional changes of tumors and organs at risk in real time, is a key technology for ensuring precise dose delivery and reducing irradiation to normal tissues [5,6,7]. In recent years, extensive research has been conducted in both clinical and engineering fields on respiratory motion management, respiratory gating, and real-time tumor tracking, promoting the in-depth development of accelerators and imaging systems [8,9,10,11,12,13].

Kilovoltage (kV) X-ray fluoroscopic image-guided radiotherapy is a common solution, yet the low soft-tissue contrast and organ occlusion often result in poor tumor visibility and blurred edges in fluoroscopic images. Clinically, it is necessary to implant fiducial markers around the tumor to assist in computer-aided tumor localization [14,15,16]. However, the implantation procedure of these markers is invasive and may lead to complications such as pneumothorax, intra-alveolar hemorrhage, and acute pneumonia [17,18,19,20]. Marker-less methods, which eliminate the need for marker implantation surgery, can avoid the associated surgical risks and complications, offering higher patient acceptance and greater potential for widespread adoption, and have thus gradually become a research hotspot. These methods often rely on temporal information and anatomical priors from imaging modalities, achieving tumor localization and trajectory estimation through strategies such as image segmentation, registration, optical flow estimation, or deep learning regression [13,21]. Segmentation-based approaches using kV X-ray images face significant challenges due to low soft-tissue contrast and the style discrepancy between real fluoroscopic images and digitally reconstructed radiograph (DRR) images used for training neural network models. These challenges severely limit segmentation performance and generalization across patients, posing a major bottleneck for clinical translation.

Benefiting from recent advances in machine learning technology and its broad application and research in the field of medical image processing [22,23,24,25], several deep learning-based marker-less tumor tracking methods have been proposed using kV X-ray fluoroscopic images. For instance, Roggen et al. [26] utilized Mask R-CNN for vertebral bone detection in acquired projection images, achieving high accuracy in spinal motion detection. He et al. [27] proposed a neural network method based on ResNetGAN, achieving sub-millimeter accuracy for marker-less motion tracking in paravertebral stereotactic body radiation therapy. However, neither method is applicable to tracking soft-tissue tumors such as those in the liver or lungs.

Among the reports on marker-less tracking suitable for lung cancer, it is common practice to train a model specifically for each individual patient. For example, Terunuma et al. [28] proposed an attention enhancement strategy combining random overlay and noise injection, which endowed the deep learning model with stronger interpretability and controllability, improving the real-time segmentation accuracy of lung tumors in kV fluoroscopic images. Their ground truth generation method was relatively complex, while the validation was primarily conducted on large tumors in the right lung, lacking coverage for cases significantly affected by heartbeats (e.g., in the left lung) or cases with smaller tumors. Zhou et al. [7] proposed using Detectron2 [29], pre-trained on the COCO dataset [30], and performed transfer learning using Gaussian-filtered DRR images. The prediction results from this patient-specific model showed a total median 3D deviation of 2.27 mm, but the tumor detection rate was not high enough. Hirai et al. [31] proposed using a deep neural network (DNN) to develop regression models for motion detection based on the image patterns of one or more regions of interest (ROIs). This method does not directly detect the tumor position and contour, thus potentially overcoming situations where the tumor is severely obscured, such as by the liver. They achieved a positional accuracy with a Euclidean distance of 1.64±0.73 mm in 10 cases involving the liver and lung. However, certain limitations remain: besides requiring training a separate DNN for each patient, manual setting of one or more ROIs in the region of maximum displacement on DRR images is needed during the training phase. Furthermore, changes in the shape and size of patient organs and tumors as treatment progresses may lead to substantial differences between the ROIs defined during training and the corresponding regions in subsequently acquired images, potentially degrading model performance. Yan et al. [32] optimized the tumor label generation and grayscale conversion algorithms to produce labels whose boundaries better fit the actual visible tumor edges and to preserve more contrast information during grayscale conversion. They used a cascaded patient-specific U-Net for tumor segmentation, localization, and tracking. This method could not handle cases with severe liver occlusion, and the overall accuracy in tumor localization and tracking was relatively low.

These studies collectively highlight the persistent challenges in utilizing kV X-ray fluoroscopy for tumor tracking, namely the poor generalizability of segmentation models across patients and the inherent difficulty of segmenting tumors from noisy images. To address these issues, we propose a novel workflow for real-time, marker-less tumor tracking designed for cross-patient applications.

**Figure 1:**
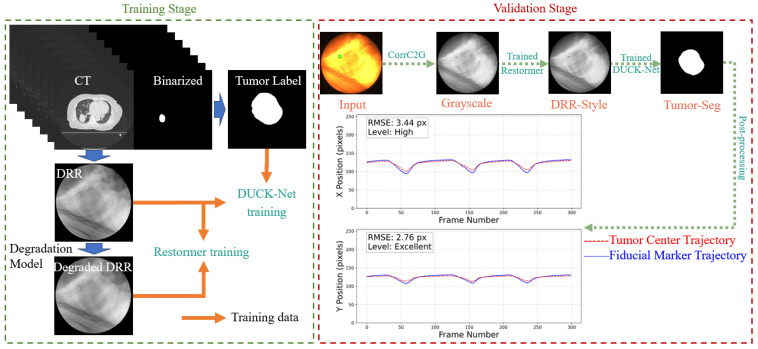
Workflow of the proposed pipeline. The green square from the input CFI represents the selected fiducial marker, which was implanted around the tumor to assist in determining the tumor location. In this study, we used the optical flow method to obtain the motion trajectory of the selected fiducial marker, which was used for comparison against the predicted tumor trajectory.

Our workflow sequentially processes the images as follows: First, color fluoroscopic images are converted to grayscale using the CorrC2G algorithm [33]. Next, a transfer-learned Restormer model transforms the image into a clean, DRR-like style. Subsequently, a DUCK-Net model performs tumor segmentation, from which the tumor’s central trajectory is extracted and smoothed via a low-pass filter (LPF). The complete pipeline is illustrated in Figure 1.

The primary objective of this study is to serve as an initial validation of this approach, evaluating its potential to achieve high tracking accuracy across a multi-patient dataset without patient-specific retraining. The core contributions of this work are (1) the proposal of a new degradation model to generate more realistic simulated training data and (2) quantitative analysis of the trade-offs between tracking accuracy and computational efficiency for the style transfer step on clinical data, providing a critical basis for its practical implementation.

The trained models and Python 3.9.19 scripts used in this study are available on GitHub (https://github.com/YongxuanYan/General_Markerless_Tumor_Tracking accesssed on 7 September 2025).

The remainder of this paper is organized as follows: Section 2 introduces the materials and methods, including data and preprocessing, model training, and post-processing. Section 3 presents the experimental results, covering evaluations of grayscale conversion and style transfer effectiveness, segmentation performance, trajectory smoothing, and overall localization accuracy. Section 4 provides a discussion of the findings, and finally, Section 5 concludes the paper.

## 2. Materials and Methods

### 2.1. Equipment and Data

The computer configuration used for algorithm development and model training is detailed in Table 1.

The real-time motion management platform for radiation therapy used in this study integrates a medical linear accelerator (TrueBeam, Varian Medical Systems, Palo Alto, CA, USA) with a real-time tumor tracking system (SyncTraX, Shimadzu Co., Kyoto, Japan). The SyncTraX system employs two pairs of kilovoltage (kV) X-ray tubes and color image intensifiers, mounted on ceiling and floor rails, respectively, enabling stereoscopic fluoroscopic imaging from various preset positions during treatment [34].

The dataset includes 4D planning CT data and CFIs from 7 lung cancer patients undergoing real-time tumor tracking radiotherapy. The tumors were located in the right lower lobe (1 case), right middle lobe (4 cases), and left upper lobe (2 cases). All patients had fiducial markers implanted; these markers served solely as a reference standard for evaluation and were not used as input for the tracking algorithm. The dataset comprises 50 pairs of consecutively acquired CFI sequences from seven different patients. Each pair consists of X-ray fluoroscopic images captured from two stereoscopic imaging viewpoints at a frequency of 30 frames per second, resulting in a total of 100 CFI image sequences.

The 4D planning CT for each patient had a slice thickness of 2 mm and covered 10 respiratory phases. For all 4D-CT datasets in this study, inter-slice mean interpolation was performed to reduce the slice thickness from 2 mm to 1 mm, thereby enhancing the fidelity of subsequent DRR generation. The tumor contours delineated by radiation oncologists across all respiratory phases were also interpolated to obtain smooth volumetric labels.

Using the interpolated 4D-CT data, DRRs and corresponding tumor label maps were generated at couch angles ranging from 0° to 355° in 5° increments, resulting in 72 couch positions per respiratory phase. The resolution of each DRR-label pair was set to 256 × 256 pixels. Tumor labels were generated using a threshold projection algorithm with a threshold of 7 voxels [32]. Subsequently, morphological opening and closing operations were applied using a 10 × 10 elliptical structuring element to eliminate small isolated white regions and fill minor black voids. In this study, DRRs and tumor labels were used to train the segmentation network. DRR rendering adhered to the same imaging geometry as the SyncTraX system to ensure spatial consistency with CFI acquisition.

To ensure spatial consistency and facilitate training, background standardization was applied to all DRRs and CFIs [32]. Specifically, a Standard Background (SB) mask was extracted, where pixel values within a circular region were set to 1 and pixels outside this region were set to 0. The same SB mask was applied to CFIs, DRRs, tumor labels, and all neural network model outputs, ensuring identical background regions to improve network convergence speed.

### 2.2. Algorithms and Neural Network Models

#### 2.2.1. Grayscale Conversion Algorithm

CorrC2G [33] is a correlation-based global color-to-grayscale conversion method. This method estimates three linear weighting parameters directly by calculating the Pearson correlation coefficients between each RGB channel and a contrast image. We selected CorrC2G for several reasons: It supports real-time processing without iterative optimization or training, and its computational complexity is controllable via a parameter *r*, which balances speed and quality. The average processing time was 3.62 ms per image in this study; CorrC2G provides two contrast mappings and automatically selects the best result via histogram analysis. It offers performance superior to most existing methods and produces natural-looking output images with preserved contrast, which aligns with human visual perception.

#### 2.2.2. Style Difference

For the tumor segmentation model, minimizing the style discrepancy between the training images and the real images is expected to enhance tumor segmentation performance of the model, thereby benefiting tumor localization and tracking accuracy. Compared to real projection images, DRR images exhibit poorer sharpness and different contrast [7]. Furthermore, real fluoroscopic images contain significant noise and blur. This noise and blur primarily stem from two factors: Firstly, the scattering of high-energy photons during propagation through tissue, causing the grayscale value of a pixel to be influenced not only by the line integral at that location but also by surrounding pixels, especially with the additional scatter from megavoltage X-rays during treatment; secondly, quantum noise and electronic noise inherent to the X-ray detector are unavoidable.

To better understand the style difference between real fluoroscopic images and DRRs, we captured multiple images of the same target, computed their average image as a clean reference, and derived noise in the images by subtracting individual frames from this average. Specifically, given a sequence of *N* fluoroscopic images $\left\{I_{1},I_{2},\ldots ,I_{N}\right\}$, the clean reference image $I_{\mathrm{avg}}$ is first computed by pixel-wise averaging:

$$I_{\mathrm{avg}}\left(x,y\right)=\frac{1}{N}\sum_{k=1}^{N}I_{k}\left(x,y\right)$$

where (x,y) denotes the pixel coordinates. Subsequently, a set of noise-only images $\left\{N_{1},N_{2},\ldots ,N_{N}\right\}$ is derived by subtracting this average from each individual frame:

$$N_{k}\left(x,y\right)=I_{k}\left(x,y\right)-I_{\mathrm{avg}}\left(x,y\right)$$

The statistical heatmaps are then generated from this set of noise images. The noise probability, $P_{N}\left(x,y\right)$, is defined as the fraction of frames in which the absolute noise value $|N_{k}\left(x,y\right)|$ exceeds a specific threshold τ. The calculation is performed using the indicator function I(·):

$$P_{N}\left(x,y\right)=\frac{1}{N}\sum_{k=1}^{N}I(|N_{k}\left(x,y\right)\left|>\tau \right),\;\mathrm{where}\;\tau =2$$

The selection of the threshold τ is a critical step that directly influences the sensitivity of noise detection. The optimal value for this threshold is contingent upon the quality of the ‘clean’ reference image, $I_{\mathrm{avg}}$. An ideal reference image would be entirely free of noise, allowing for a very low threshold. However, in practice, an image averaged from a finite number of frames always retains some residual structural or random noise. If this residual noise in $I_{\mathrm{avg}}$ is significant, i.e., the reference image itself appears grainy, a low threshold τ would erroneously classify these baseline fluctuations as significant temporal noise events, leading to an overestimation of noise across the entire image. Therefore, a noisier reference image necessitates a higher threshold to avoid false positives. Here, the reference image was generated by averaging a large set of 1800 frames. This large sample size ensures a very high signal-to-noise ratio and effective suppression of random fluctuations, resulting in a very clean and stable baseline. Consequently, a relatively low threshold of τ=2 was sufficient to capture genuine noise events without misinterpreting the baseline of the reference image.

The noise amplitude, $A_{N}\left(x,y\right)$, is quantified by the mean absolute deviation of the noise values across all frames:

$$A_{N}\left(x,y\right)=\frac{1}{N}\sum_{k=1}^{N}\left|N_{k}\left(x,y\right)\right|$$

**Figure 2:**
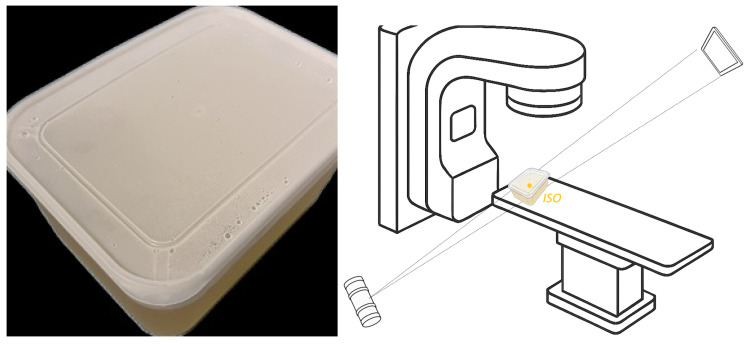
Gelatin cube preparation and image acquisition: (**left**) container with gelatin inside; (**right**) schematic diagram of image acquisition site, the gelatin container was placed at the ISO center.

**Figure 3:**
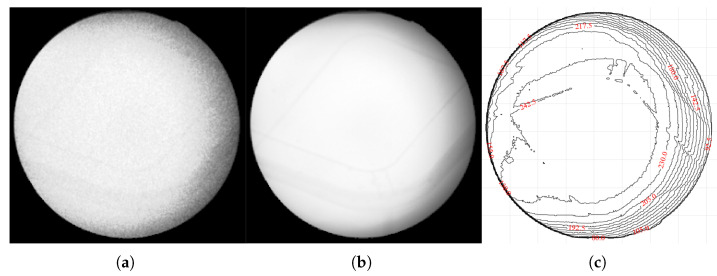
Captured X-ray images of the gelatin cube: (**a**) an X-ray fluoroscopic image captured by the SyncTraX system, grayscaled using CorrC2G; (**b**) clean image calculated by averaging 1800 images; (**c**) brightness contour map of the average image. The brightness contour map (**c**) shows that the brightness center of the image is shifted slightly to the left and does not overlap with the center of the image, which indicates that the central axis of the X-ray beam is offset. Since the gelatin container was placed at the ISO center and its shape is not circular, it is unlikely that this brightness deviation is caused by the container.

**Figure 4:**
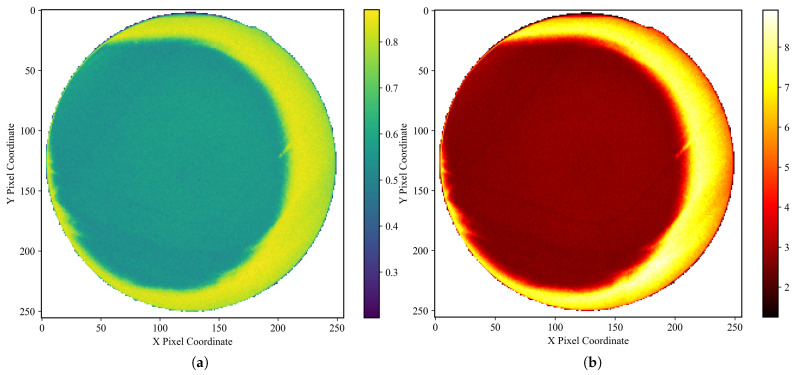
Noise characteristics in a gelatin cube fluoroscopic image (background pixels excluded). (**a**) Heatmap of degradation-induced noise probability and (**b**) amplitude, derived by computing the pixel-wise variance across 1800 subtracted images (individual frames minus the average of all frames) and normalizing it to a [0,1] range. In (**a**), the mean noise probability within the central circular region is approximately 0.56, while that in the right peripheral arc region reaches about 0.83. In (**b**), the mean noise amplitude in the central circular region is approximately 2.7, increasing to around 7.2 in the right peripheral arc. Regions with lower grayscale values in the image, such as the cube’s edges, exhibit lower noise probability and amplitude, and the farther the area is from the center of the X-ray beam (note: the beam center, not the image center), the higher the noise probability and amplitude. Importantly, the empirical profile is not strictly monotonic: noise probability and amplitude increase with radial distance from the beam center until approximately one radius, after which a decline is observed. This decline becomes noticeable primarily on the right-hand side of the image, due to a slight leftward shift of the X-ray beam center, which exposes more of the far-right region beyond the effective beam axis. Here, the radius refers specifically to the radius of the circular foreground region in the image.

**Figure 5:**
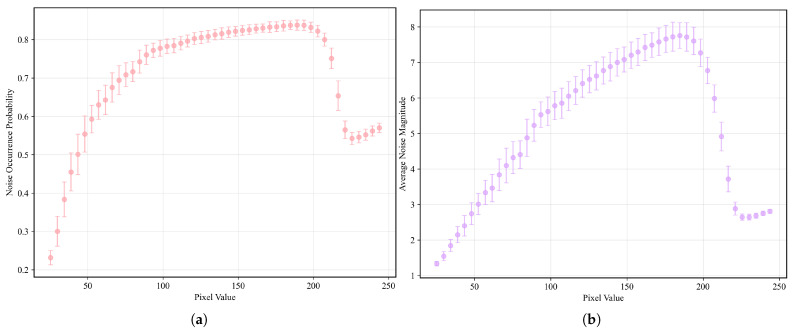
(**a**) Relationship between pixel grayscale value and noise probability. (**b**) Relationship between pixel grayscale value and noise amplitude. From a holistic perspective, the relationship can be broadly divided into four phases: rapid ascent phase (pixel values between 0 and 85), slow ascent phase (pixel values between 85 and 190, the average noise probability and amplitude are around 0.81 and 6.5, respectively), sharp decline phase (pixel values between 190 and 225), and recovery phase (pixel values between 225 and 255, the avearge noise probability and amplitude are around 0.56 and 2.75, respectively). The sharp decline phase is likely attributable to the clipping of high-amplitude noise fluctuations that exceed the upper grayscale limit of 255, while the recovery phase corresponds to the re-elevation of the overall noise level by low-amplitude noise. Given the presence of various types of noise [35,36] in X-ray imaging—such as quantum noise, electronic noise and structural noise from the imaging device—which exhibit different frequencies and amplitudes, this may account for the observed four-phase relationship.

This approach allows for a direct, empirical characterization of the noise’s frequency and typical magnitude at each pixel location. To ensure that the target remained motionless between frames, we fabricated a gelatin cube and acquired 1800 X-ray fluoroscopic images under 100 kV, 63 mA, and 4 ms conditions using the SyncTraX system (SMART version), as illustrated in Figure 2 and Figure 3. The results of the statistical analysis of the subtracted noise images are shown in Figure 4 and Figure 5. Our analysis revealed that the noise probability and amplitude in real X-ray fluoroscopic images are not globally random but exhibit systematic correlations with both pixel location and intensity values.

Simulating these physical characteristics enables us to generate simulated fluoroscopic images from DRRs that possess noise and blur similar to real fluoroscopic images. This allows us to use paired image data of DRRs and simulated images to train a neural network model, equipping it with the ability to transform real X-ray fluoroscopic images into the DRR image style. To this end, we designed a DRR degradation modeling method, primarily including a Gaussian mist scattering model and a content- and spatially dependent noise model.

#### 2.2.3. Degradation Modeling

To accurately simulate the complex fluoroscopic image degradation style observed in clinical settings, we propose a stochastic degradation model. This model explicitly accounts for both the non-uniform, signal-dependent noise and the inherent diffusion caused by physical and electronic effects. The degradation process is composed of several carefully designed stages to generate highly realistic simulated images.

##### Noise Characteristic Functions

The noise characteristics at any pixel p=(x,y) are defined by the noise probability ($N_{p}$) and the noise amplitude ($N_{a}$) maps. These maps are calculated as follows:

(1) $$N_{p}\left(V,d\right)=\mathrm{scale}\left(P_{\max},B_{p},f_{vp}\left(V\right)\cdot f_{sp}\left(d,X_{c},\sigma_{d}\right)\right)$$

(2) $$N_{a}\left(V,d\right)=\mathrm{scale}\left(M_{\max},B_{a},f_{va}\left(V\right)\cdot f_{sa}\left(d,X_{c},\sigma_{d}\right)\right)$$

where *V* is the pixel intensity value. $P_{\max}$ and $M_{\max}$ are the upper limits for noise probability and amplitude, respectively, which are set to 0.92 and 16 based on experimental observations. $f_{vp}\left(\cdot \right)$ and $f_{va}\left(\cdot \right)$ are functions describing the content dependency of noise. $f_{sp}\left(\cdot \right)$ and $f_{sa}\left(\cdot \right)$ are functions describing the spatial dependency of noise. $\sigma_{d}$ is the central flatness radius parameter. $X_{c}=\left(x_{c},y_{c}\right)$ denotes the random offset of the X-ray beam center $d_{\mathrm{beam}}$ from the image geometric center $d_{\mathrm{img}}$:

(3) $$X_{c}=d_{\mathrm{beam}}-d_{\mathrm{img}}$$

and *d* is the radial distance from the current pixel p to the actual beam center:

(4) $$d={∥p-d_{\mathrm{img}}-X_{c}∥}_{2}$$

The function scale(max,min,·) represents a linear mapping of the raw calculated noise characteristic to a target range defined by the base value ($B_{p}$ or $B_{a}$) and the maximum value ($P_{\max}$ or $M_{\max}$).

##### Image Content Dependency

**Figure 6:**
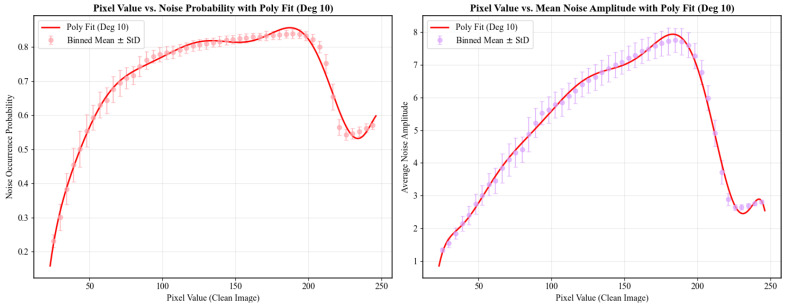
Fitting results using a 10th-order polynomial.

The content dependency functions $f_{vp}\left(V\right)$ and $f_{va}\left(V\right)$ capture the complex, multi-phase relationship between pixel intensity and noise level. To accurately represent the observed non-linear trends (e.g., rapid ascent, slow ascent, sharp decline, and recovery), we fit a 10th-order polynomial to curves smoothed from experimental data, as illustrated in Figure 6. In our implementation, the output of $f_{va}\left(V\right)$ is multiplied by a factor of 2 to match empirical data.

##### Spatial Dependency

The spatial dependency term models the phenomenon of minimum noise at the beam center, which gradually increases towards the periphery. The spatial functions $f_{sp}$ and $f_{sa}$ are defined as follows:

(5) $$f_{s\zeta }\left(d,X_{c},\sigma_{d}\right)=B_{\zeta }+F_{d}\left(d,\sigma_{d}\right)$$

where ζ∈{p,a}, and $B_{\zeta }$ is the base noise level (either probability $B_{p}$ or amplitude $B_{a}$), ensuring a baseline level of noise even at the center. $F_{d}\left(\cdot \right)$ is the core radial modification function:

(6) $$F_{d}\left(d,\sigma_{d}\right)=1-\exp\left(-\frac{d^{2}}{2\sigma_{d}^{2}}\right)$$

##### Noise Generation and Diffusion

Instead of direct noise addition, our model employs a multi-step process to simulate the physical diffusion properties of noise, as detailed in Algorithm 1.
**Algorithm 1** Stochastic Noise Generation and Diffusion ProcessInput: Original DRR image $I_{DRR}$.Output: Degraded Image $I_{deg}$.Generate a uniform random matrix R∼U(0,1) of the same size as $I_{DRR}$.Create a binary noise mask *M* where M(p)=1 if $R\left(p\right)<N_{p}\left(p\right)$.Initialize an empty noise array $I_{noise\_init}$.Loop for each pixel p where M(p)=1.Sample value $v∼N(\mu ,\sigma =N_{a}\left(p\right))$ and assign to $I_{noise\_init}\left(p\right)$, where μ is the global noise mean.End loop.Apply a Gaussian filter G(·) with σ=2.0 to the sparse noise array:         $I_{noise\_blur}=G\left(I_{noise\_init}\right)$.Blend the original image with the blurred noise:         $I_{blend}=I_{DRR}+I_{noise\_blur}$.Generate a secondary noise array $I_{noise\_sec}$ by sampling from N(μ,σ) at locations defined by the mask *M*.Add the secondary noise to the blended image:          $I_{deg}=I_{blend}+I_{noise\_sec}$.Clip pixel values of $I_{deg}$ to the range [0,255] and convert to 8-bit integer.Return $I_{deg}$.

##### Stochastic Strategy for Generalization

Stochastic variables are introduced into the key parameters of the model, as shown in Table 2. By resampling these stochastic variables during the generation of each degraded image, the simulated data achieves high diversity and is expected to simulate from different devices and acquisition settings, thereby enhancing the generalization performance of the Restormer model.

**Figure 7:**
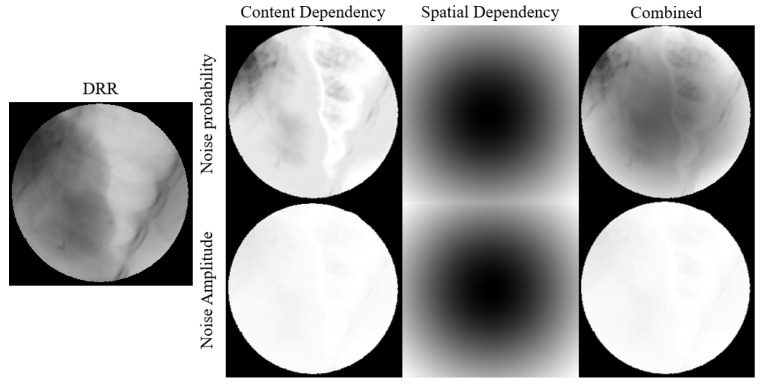
Visual representation of how pixel values and spatial position relationship affects noise probability and amplitude.

The noise probability and amplitude generated using our modeling method are illustrated in Figure 7.

#### 2.2.4. Transfer Learning of Restormer

To mitigate the domain gap between CFI and DRR, this study employs Restormer [37] for style transfer. Restormer is an efficient Transformer model specifically designed for high-resolution image restoration tasks. Its core innovation lies in the redesign of the traditional Transformer modules, namely the multi-head self-attention and feed-forward network, which significantly reduces computational complexity while retaining global modeling capability. The choice of Restormer is motivated by its several key advantages: it enables efficient high-resolution image processing through its multi-dconv head transposed attention module, which reduces computational cost by shifting attention computation from spatial to channel dimensions; it effectively integrates global and local contexts by combining the global modeling capability of the Transformer with local details captured through depthwise convolution, thereby enhancing restoration quality while preserving fine details; it exhibits superior cross-task generalization, having achieved state-of-the-art performance across multiple restoration tasks such as image deraining, deblurring, and denoising; it supports a progressive training strategy that improves the modeling of global image statistics; and its lightweight design, with fewer parameters and lower computational cost compared to other Transformer models like IPT and SwinIR, offers faster inference speed and higher practicality.

To fully leverage its prior knowledge, we initialize the Restormer model with publicly available Real Image Denoising (RealDenoising) pre-trained weights released by the authors.

The input consists of simulated fluoroscopic-style images generated using the degradation model proposed in Section 2.2.3, while the target output is the corresponding clean DRR. This paired design enforces the network to learn the mapping from degraded inputs to DRR-style outputs, thereby narrowing the gap between the two domains. We trained a baseline version by applying globally random Gaussian noise. The total training epochs are 345; except for the configurations mentioned in Table 3, all other training details for the Restormer model remain the same as those specified by the authors.

After fine-tuning, Restormer is integrated as part of the preprocessing pipeline, transforming CorrC2G-converted grayscale fluoroscopic images into DRR-style images, which are then fed into DUCK-Net for tumor segmentation.

### 2.3. Tumor Segmentation Network

To segment the tumor region from the output of the Restorer, we employed DUCK-Net [38], an encoder–decoder convolutional neural network specifically designed for medical image segmentation, particularly for colon polyp segmentation. DUCK-Net was chosen as the polyp segmentation model due to its combination of powerful multi-scale feature fusion, exceptional generalization capability, and practical efficiency. The model’s DUCK block captures both local details and global context through a parallel multi-path architecture, enabling it to effectively handle polyps of diverse sizes, shapes, and textures. Notably, DUCK-Net achieves state-of-the-art performance on multiple public datasets without relying on external pre-training, unlike many Transformer-based approaches. Its robustness is further enhanced by a strong data augmentation strategy implemented via the Albumentations library, which improves adaptability to real-world variations in lighting, angle, and scale. In addition, the model is computationally efficient, featuring fewer parameters and faster inference compared to Transformer-based alternatives, while its multi-path convolutional design offers strong interpretability, yielding well-defined segmentation masks with reduced false positives. DUCK-Net also demonstrates excellent generalization in cross-dataset tests, maintaining high accuracy even when trained and tested on data from different sources. This reliability under varying clinical conditions underscores its strong practical applicability, making it particularly suitable for lung tumor segmentation, where tumor appearance and scale can vary significantly.

#### 2.3.1. Network Input and Configuration

Since the output from the previous Restormer stage is a grayscale image, the input to DUCK-Net is a single-channel image with a uniform size of 256×256 pixels. The network outputs a binary tumor mask with the same spatial resolution as the input. The number of convolutional filters in the initial stage was set to 17.

#### 2.3.2. Training Settings

The training was conducted for 700 epochs. The initial learning rate $\eta_{0}$ was set to $2\times 10^{-3}$, decaying by $1\times 10^{-5}$ after each epoch until reaching a minimum value of $1\times 10^{-5}$:

(7) $$\eta_{t+1}=\max\left(10^{-5},\eta_{t}-10^{-5}\right),\;\;\;\left(t\geq 0\right).$$

Most parameter settings were consistent with those in the original DUCK-Net paper. Specifically, optimization was performed using the Adam optimizer with $\beta_{1}=0.9$, $\beta_{2}=0.999$, and weight decay of $10^{-4}$. A fixed random seed was used when splitting training, validation and test datasets to ensure reproducibility. Data augmentation included horizontal and vertical flipping, random affine transformations (scaling factor 0.5–1.5, translation range ±12.5%, rotation range $\pm 180^{\circ }$, shear range $\pm 22.5^{\circ }$), and color jitter (brightness range 0.6–1.6, contrast range ±0.2, saturation range ±0.1, hue range ±0.01). We additionally incorporated Gaussian blur and Gaussian noise to handle cases where Restormer failed to achieve complete DRR stylization. All augmentation operations were applied with a probability of 1.0, except for Gaussian blur and Gaussian noise, which were applied with a probability of 0.5. The loss function remained the same as in the original paper, using Dice Loss.

### 2.4. Trajectory Extraction and Post-Processing Analysis

Due to the lack of ground truth tumor labels for evaluation of the segmentation output, we extracted the temporal trajectory of the tumor via centroid calculation and compared it with the motion trajectory of the fiducial markers to assess the tumor segmentation performance. For the segmentation result $\Omega_{t}\subset R^{2}$ at time *t*, the tumor center position $\left(x_{t},y_{t}\right)$ is defined as follows:

(8) $$x_{t}=\frac{1}{|\Omega_{t}|}\sum_{\left(i,j\right)\in \Omega_{t}}i,\;y_{t}=\frac{1}{|\Omega_{t}|}\sum_{\left(i,j\right)\in \Omega_{t}}j,$$

where $|\Omega_{t}|$ denotes the number of tumor pixels at time *t*, and (i,j) represents the pixel coordinates. Using this method, we obtain the complete trajectory sequence:

(9) $$T={\left(x_{t},y_{t}\right)}_{t=1}^{N},$$

where *N* is the number of frames in the sequence; in this study, images were collected at a rate of 30 frames per second, so the relationship between time *t* and *N* is: t=N/30 s.

#### 2.4.1. Low-Pass Filtering

As the megavoltage (MV) level X-ray scatter noise during radiation therapy can affect tumor segmentation results in some frames, the extracted motion trajectory sequence often contains high-frequency jitter components. This study employs a fourth-order Butterworth low-pass filter to smooth the trajectory. This is very important for systems that determine whether the tumor has reached the beam output position based on detected coordinates. The digital filter was designed based on the following parameters: cutoff frequency $f_{c}=8$ Hz, sampling frequency $f_{s}=30$ Hz. Its transfer function in the continuous-time domain is expressed as follows:

(10) $$H\left(\omega \right)=\frac{1}{\sqrt{1+{\left(\frac{\omega }{\omega_{c}}\right)}^{2n}}},$$

where $\omega_{c}=2\pi f_{c}$ is the angular cutoff frequency and n=4 is the filter order. In the practical digital implementation, it was discretized using the bilinear transform, with the corresponding normalized cutoff frequency calculated as $\omega_{\mathrm{norm}}=f_{c}/\left(0.5\times f_{s}\right)$.

**Figure 8:**
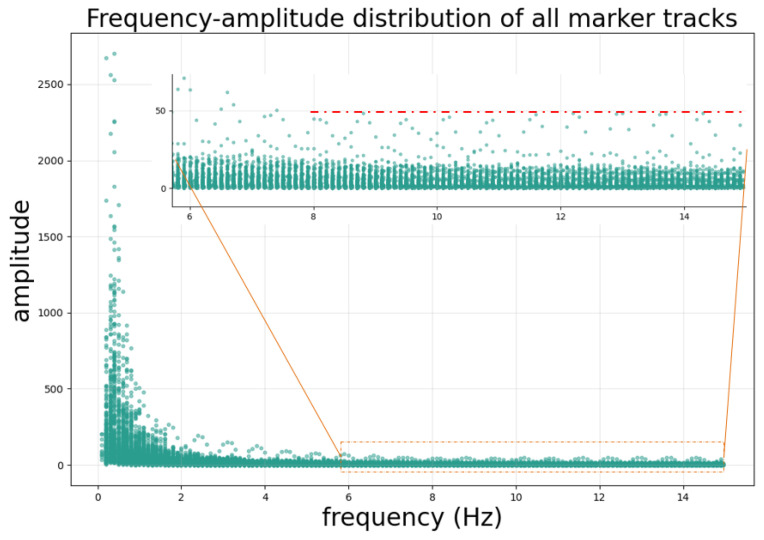
Frequency–amplitude distribution of the fiducial marker motion trajectory. Frequency analysis reveals the typical harmonic structure of respiratory motion, with a fundamental frequency of approximately 0.33 Hz (20 breaths/min) and multiple harmonics extending up to 15 Hz. Notably, the amplitude of harmonic components above 8 Hz remains stable at a low level (amplitude below 50 as indicated by the red dashed line). Based on this observation, we selected 8 Hz as the cutoff frequency to retain physiologically relevant motion components while filtering out trajectory jitter introduced by segmentation errors in individual frames, which corresponds to the high-frequency components.

A global frequency analysis was performed on all reference marker trajectories to accurately characterize the frequency components of the motion signals. By applying the Fast Fourier Transform, the frequency spectra and corresponding amplitudes were computed. The frequency and amplitude data from all trajectories were then aggregated to generate a frequency–amplitude scatter plot, as illustrated in Figure 8. The motion of the fiducial markers or the tumor center point is primarily influenced by the patient’s respiration and heart rate, with the frequency energy concentrated mainly below 2 Hz. This study selected a cutoff frequency of 8 Hz to filter the motion trajectory of the tumor center point.

The filtering operation was implemented using a zero-phase filtering method to avoid introducing phase distortion, ensuring that the filtered trajectory remains precisely aligned in time with the original data.

#### 2.4.2. 3D Motion Trajectory Comparison

**Figure 9:**
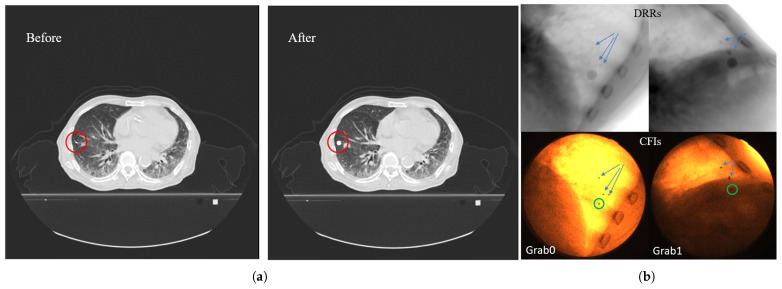
Fiducial marker expansion and matching method: (**a**) Illustration of the fiducial marker expansion process in CT data: the left panel shows the original CT slice with a red circle highlighting the target fiducial marker; the right panel shows the same slice after expanding the marker region into a spherical volume (5 mm radius, 3000 HU). (**b**) Fiducial marker matching between DRR and clinical fluoroscopic image: the upper row shows DRR images generated with identical geometric parameters to the actual imaging setup; the lower row shows the corresponding clinically acquired CFIs. The green circles indicate the identified target fiducial marker in the CFIs. Arrows in both DRR and CFIs point to other surrounding fiducial markers, demonstrating successful spatial correspondence between the expanded marker region in DRRs and the actual marker position in CFIs.

As introduced in the equipment section, the stereoscopic imaging system consists of two pairs of imaging devices acquiring two image sequences simultaneously. Since identifying and pairing the same fiducial marker across images from these two different imaging systems is challenging by visual inspection, we developed a method to determine the position of the selected fiducial marker from images captured under different imaging positions. This paper proposes a fiducial marker dilation method for marker matching, illustrated in Figure 9. The method involves three steps: (1) A suitable fiducial marker is selected from the CT data. The selection principle is to choose one as close to the tumor as possible and sufficiently distant from other markers to avoid overlap or proximity issues in certain angles. (2) Dilating selected marker: set all voxels within a 5 mm radius sphere centered on the marker’s center voxel to 3000 Hounsfield Units (HUs). This process is akin to inflating the marker into a sphere with a diameter of 1 cm. (3) DRR Generation: DRRs using geometric parameters identical to those of the acquired X-ray fluoroscopic images are then generated.

In the generated DRR images at any angle, the selected marker region appears as a circular area, with its center indicating the marker’s position. By comparing the DRR with the actual acquired fluoroscopic image and using nearby bone structures, tissues, and the relative positions to other markers as references, the position of the selected fiducial marker in the real X-ray fluoroscopic image can be determined.

For markers that remain difficult to identify, one can either select a different marker or reduce the dilation volume for confirmation. Subsequently, optical flow is used to track the target fiducial marker. The 3D spatial motion trajectory is then calculated using the imaging geometry information and the marker’s coordinates in the images.

To accurately compare the motion trajectories of the tumor center point and the fiducial marker, it is essential to analyze their relative movement while eliminating the constant spatial offset arising from their non-overlapping initial positions in the image plane. A direct comparison of the raw coordinates would introduce a large baseline error, even if their motion patterns were identical. Therefore, we aligned the trajectories by normalizing the coordinate system to the average position of the fiducial marker. This alignment removes the global displacement component caused by imaging geometry, ensuring that the comparison focuses solely on the dynamic motion relative to a common baseline.

The alignment is performed using the following equations:

(11) $$x_{t}^{\prime }=x_{t}-\left(\frac{1}{N}\sum_{t=1}^{N}x_{t}-\frac{1}{N}\sum_{t=1}^{N}x_{t}^{m}\right),\;y_{t}^{\prime }=y_{t}-\left(\frac{1}{N}\sum_{t=1}^{N}y_{t}-\frac{1}{N}\sum_{t=1}^{N}y_{t}^{m}\right).$$

In these equations, $x_{t}$ and $y_{t}$ represent the raw 2D image coordinates of the tumor center point at frame *t*, while $x_{t}^{m}$ and $y_{t}^{m}$ represent the corresponding coordinates of the fiducial marker. The total number of frames in the sequence is denoted by *N*. The terms $\frac{1}{N}\sum_{t=1}^{N}x_{t}$ and $\frac{1}{N}\sum_{t=1}^{N}y_{t}$ calculate the mean position of the tumor center point over the entire sequence, and similarly, $\frac{1}{N}\sum_{t=1}^{N}x_{t}^{m}$ and $\frac{1}{N}\sum_{t=1}^{N}y_{t}^{m}$ calculate the mean position of the fiducial marker, which serves as the reference baseline. The resulting coordinates $x_{t}^{\prime }$ and $y_{t}^{\prime }$ are the aligned coordinates of the tumor center point. The key operation involves subtracting the constant offset—the difference between the average positions of the tumor and the marker—which effectively centers the tumor’s trajectory around the marker’s average position.

The registered trajectory $T^{\prime }=\left\{\left(x_{t}^{\prime },y_{t}^{\prime }\right)\right\}$ is then filtered using a low-pass filter. The 3D motion trajectory of the tumor center point is calculated from this aligned 2D trajectory and compared frame-by-frame with the reference trajectory derived from the fiducial marker.

#### 2.4.3. 2D Motion Trajectory Comparison

In addition to comparing 3D motion trajectories, we also performed 2D motion trajectory comparisons. This analysis focused on the in-plane motion of both the tumor center point and the fiducial marker, which was conducted to facilitate a direct comparison with a previous study [32]. Trajectory accuracy was assessed using the Root Mean Square Error (RMSE):

(12) $${\mathrm{RMSE}}_{x}=\sqrt{\frac{1}{N}\sum_{t=1}^{N}{\left(x_{t}^{\prime }-x_{t}^{m}\right)}^{2}},\;{\mathrm{RMSE}}_{y}=\sqrt{\frac{1}{N}\sum_{t=1}^{N}{\left(y_{t}^{\prime }-y_{t}^{m}\right)}^{2}}.$$

The final error *E* is taken as the maximum of the errors in the two directions:

(13) $$E=\max({\mathrm{RMSE}}_{x},{\mathrm{RMSE}}_{y}).$$

A grading standard based on the final error *E* was established, as shown in Table 4.

## 3. Results

### 3.1. Image Grayscaling and DRR Style Transfer Results

**Figure 10:**
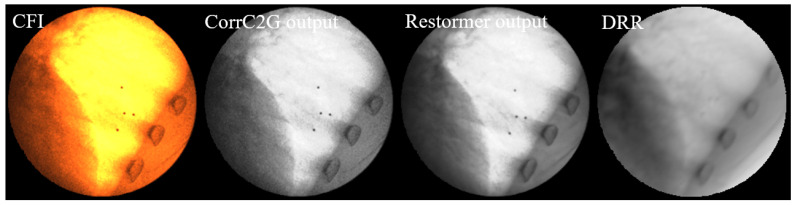
Comparison of image processing results at different stages. From (**left**) to (**right**): Original CFI; CorrC2G output; Restormer output; DRR: generated with identical imaging geometry. The CorrC2G algorithm preserves contrast information while Restormer effectively suppresses noise and blur, producing image characteristics closer to those of DRR images.

Figure 10 shows a comparison between the input CFI, the gray fluoroscopic image (GFI) processed by the CorrC2G algorithm, the output of Restormer (GFI_DRR_Style), and the DRR image generated under the same imaging geometry configuration as the CFI. It can be observed that the CorrC2G algorithm preserves the contrast information in the image. Visually, compared to the original grayscale fluoroscopic image, Restormer suppresses noise and blur in the GFI and brings the image style closer to that of the DRR image.

**Figure 11:**
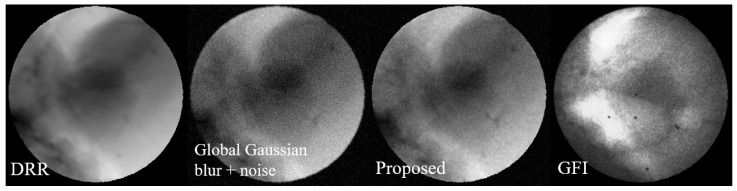
Visualization comparison of the image degradation model. From (**left**) to (**right**): DRR, degraded image with Gaussian noise, degraded image using the proposed method, and GFI. It can be seen that the degraded image generated by the proposed method can better simulate the noise pattern in GFI. It is notable that while the noise density is visibly reduced in the two highlighted areas of the GFI, the corresponding areas in our generated image, although also showing reduced noise, appear comparatively noisier. This discrepancy is because the corresponding areas in the source DRR are not as bright as those in the GFI, leading to a different noise expression after degradation.

Figure 11 shows the visual comparison using the proposed image degradation model and commonly used image augmentation methods.

**Figure 12:**
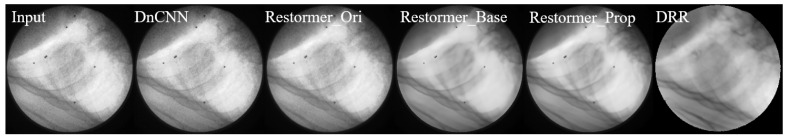
Visual comparison of denoising results using different methods on a sample X-ray fluoroscopic image. From (**left**) to (**right**): Grayscale fluoroscopic image (Input); Output from pre-trained DnCNN; Output from pre-trained Restormer (Restormer_Ori); Output from Restormer fine-tuned on baseline dataset (Restormer_Base); Output from Restormer fine-tuned on our proposed dataset (Restormer_Prop); Reference DRR. The results demonstrate that output from Restormer fine-tuned on our proposed dataset has better visual quality and closer resemblance to DRR-style images.

Figure 12 presents a visual comparison of denoising results, showing that the output images from the Restormer trained on our proposed dataset exhibit better visual quality and closer resemblance to real X-ray fluoroscopic images.

**Figure 13:**
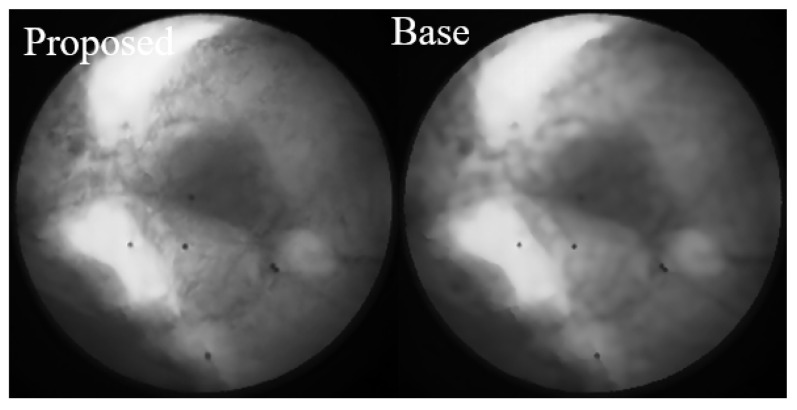
Focused comparison between the baseline method and our proposed approach. While both methods demonstrate effective noise suppression, the Restormer fine-tuned on our proposed dataset (**left**) preserves significantly more image details compared to the baseline method (**right**). This enhanced detail preservation contributes to better visual quality and a more accurate representation of anatomical structures.

As highlighted in Figure 13, we conducted a focused comparison between the baseline method and our proposed approach. While both methods demonstrate excellent noise suppression capabilities, the Restormer fine-tuned on our proposed dataset preserves significantly more image details, leading to superior visual fidelity and a more accurate representation of anatomical structures.

### 3.2. DUCK-Net Segmentation Results and Motion Trajectory Accuracy Assessment

**Figure 14:**
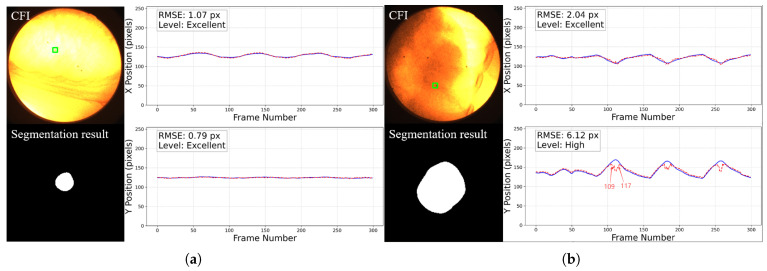

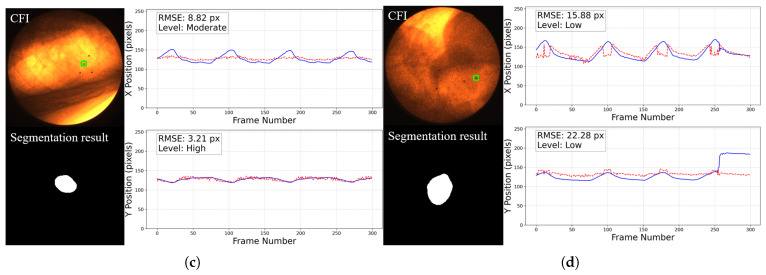
Representative cases of 2D motion trajectory comparison across different accuracy grades (LPF not applied). For each case: (**Left**) panel shows the workflow input CFI with a green square indicating the selected fiducial marker (**top**) and corresponding tumor segmentation result (**bottom**); (**Right**) panel displays the aligned temporal trajectories of tumor center (red dotted line) and fiducial marker (blue line) in image coordinates (x and y directions): (**a**) Excellent cases (50%) have near-perfect trajectory alignment. (**b**) High-accuracy cases (46%) show certain deviations in some frames (such as frame 109 to 117, we will discuss about it later) but the trajectory remains roughly aligned with the reference marker. This is common in cases where the image contrast is too poor, with severe noise and large tumor motion amplitude. (**c**) Moderate cases (3%) exhibit larger errors. (**d**) The only Low-graded case (1%) is caused by two factors: significant trajectory divergence in inspiratory frames and marker tracking error in the last 44 frames. The overall tumor detection rate is 100%.

For the 2D motion trajectory comparison, representative cases before trajectory post-processing from each grade are presented in Figure 14. In each figure, the left part displays the workflow input CFI with a green square showing the selected fiducial marker on the top and the corresponding tumor segmentation result at the bottom, and the right part shows the aligned x and y coordinate values over time for the tumor center and the fiducial marker within the image plane.

**Figure 15:**
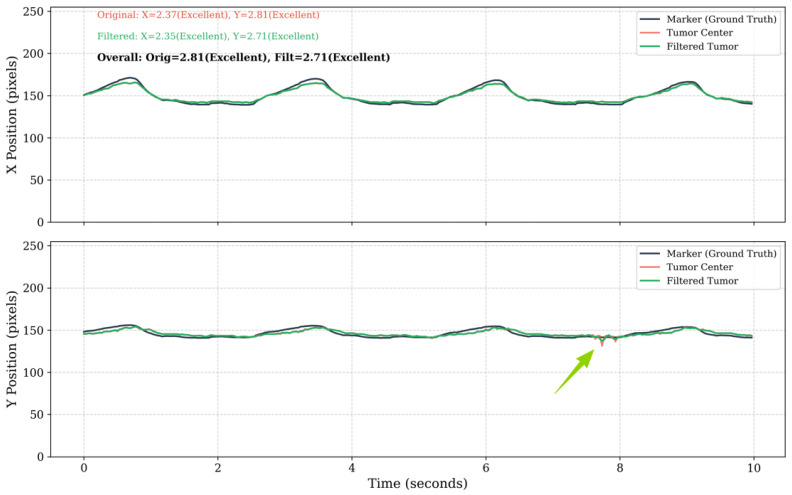
Comparison of filtered and unfiltered tumor trajectories. Unfiltered trajectory showing high-frequency jitter from segmentation error (red); Low-pass filtered trajectory (8 Hz cutoff) preserving the respiratory motion pattern while suppressing the jitter. The trajectory using the low-pass filter deviates less at the points indicated by the arrow.

With LPF post-processing, the performance is further improved (average final error *E* dropped by 0.11), as illustrated in Figure 15.

Compared to the results of previous studies, the proportion of cases achieving *Excellent* and *High* grades with the workflow used in this study increased significantly, while the number of *Moderate* and *Low* grade cases substantially decreased, as shown in Table 5. Table 6 shows the comparison of 3D tumor tracking accuracy between different methods.

**Figure 16:**
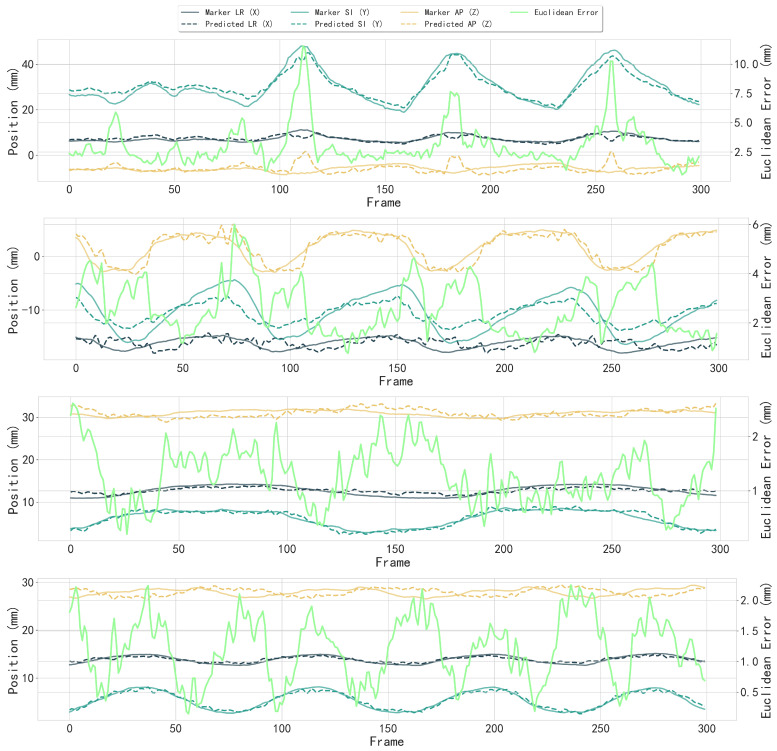
Partial results of 3D motion trajectory comparison between the fiducial marker (reference) and the tracked tumor center. Each subfigure shows individual trajectory components in LR (X), SI (Y), and AP (Z) directions with fiducial marker and detected tumor center; Euclidean distance error (bright green, right *y*-axis) between fiducial marker and tumor center across frames (*x*-axis). The median Euclidean error in all cases was 1.53 mm, with directional errors of 0.98±0.70 mm (LR), 1.09±0.74 mm (SI), and 1.34±0.94 mm (AP).

Partial visual results of the 3D motion trajectory using the proposed whole framework are shown in Figure 16.

## 4. Discussion

The non-uniform noise modeling approach proposed in this study is designed to maximize its generalizability across different imaging devices and acquisition settings. Although the primary parameterization in this research relies on data from one specific device, preliminary noise analysis conducted on a newer generation SyncTraX system provides important corroboration for the adaptability of the model.

**Figure 17:**
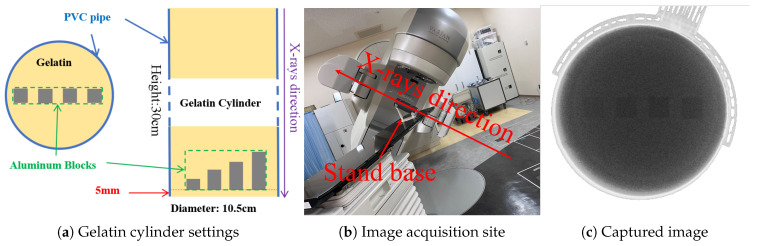
Gelatin preparation and image acquisition. The aluminum blocks are labeled `aluminum_1’ to `aluminum_4’ from left to right, with heights of 1, 2, 3, and 4 mm, respectively.

**Figure 18:**
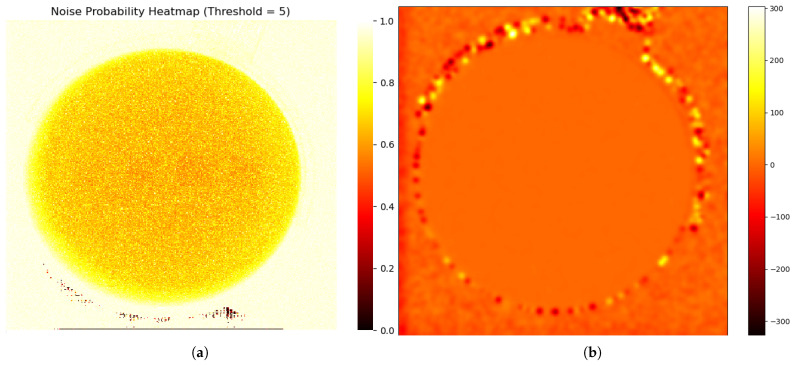
Noise characteristics in a gelatin cylinder fluoroscopic image: (**a**) Probability distribution map of degradation-induced noise, derived by computing the pixel-wise variance across 300 subtracted images (individual frames minus the average of all frames) and normalizing it to a [0, 1] range, where higher values indicate greater degradation likelihood. To account for the fact that the average image is not perfectly clean, a threshold of 5 grayscale values was applied, such that only differences exceeding this threshold were considered as noise. Square regions with embedded aluminum blocks show lower probabilities than gelatin areas, decreasing toward the image center within the circular field. (**b**) Heatmap of average noise amplitude across the image, calculated as the mean absolute difference between individual frames and the average image. The noise amplitude remains stable inside the gelatin area.

**Figure 19:**
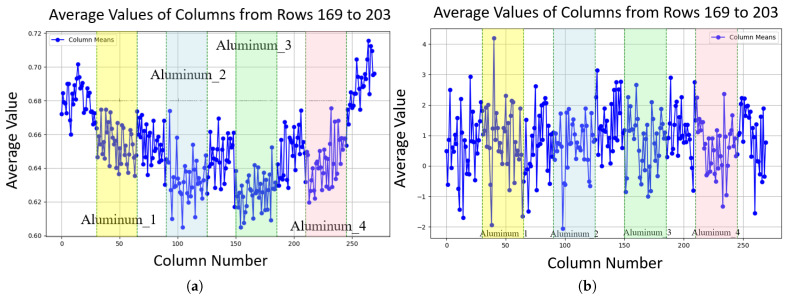
Line plots of noise characteristics along image columns, with edge pixels excluded to focus on internal variations: (**a**) Average probability along columns (rows 169 to 203 contain aluminum blocks); aluminum block regions exhibit lower probability (around 0.015 to 0.02 lower, depending on how close the pixel is to the center) compared to gelatin areas. (**b**) Average amplitude along columns (rows 169 to 203 contain aluminum blocks), showing stable amplitude in aluminum block regions compared to gelatin areas.

We fabricated a gelatin cylinder and acquired 300 X-ray fluoroscopic images under 100 kV, 80 mA, and 4 ms conditions using the newer SyncTraX system. We embedded four aluminum blocks of different thicknesses inside the gelatin to simulate bones, as shown in Figure 17. Statistical analysis results of the subtracted noise images are shown in Figure 18 and Figure 19.

In the newer device, the characteristics of noise probability ($N_{p}$) were found to be highly consistent with our primary findings: $N_{p}$ remains lower in regions of low material density (high pixel values) and gradually increases with distance from the X-ray beam center. However, a notable difference was observed in the noise mean amplitude ($N_{m}$). This contrast is evident when comparing the noise amplitude characteristics of the older device shown in Figure 5b, where the amplitude is clearly influenced by pixel grayscale values, with those of the newer device shown in Figure 19b. In the newer device, the noise amplitude exhibited uniformity across the entire image area; no significant variation in amplitude was observed between the regions corresponding to the aluminum blocks and the surrounding gelatin area. This indicates that the noise amplitude in the newer system has little sensitivity to changes in the pixel value *V* or the radial distance *d*.

This uniform amplitude characteristic is likely a result of the newer device employing improved X-ray collimating technology, which ensures highly uniform X-ray intensity, thereby eliminating the spatial intensity variations typical of older systems. For devices exhibiting such high amplitude uniformity, our proposed noise model demonstrates adaptability: the $\sigma_{d}$ and β within the amplitude functions $f_{sm}\left(\cdot \right)$ in Equation (6) can be adjusted to be larger until the $f_{sm}\left(\cdot \right)$ become insensitive to changes in *d*. This principle is analogous to the concept that “the Earth is large enough that the local terrain appears flat,” demonstrating the model’s flexible capacity. However, this finding also confirms that a limitation of our proposed modeling method is that it requires a device-specific calibration prior to its application.

To guide future adoption, this calibration procedure can be formalized into the following steps: (1) Acquire a series of images of a static phantom (e.g., a gelatin or stationary phantom) under the desired imaging conditions. (2) Calculate the mean of these images to obtain a “clean” average image, which is then subtracted from the individual frames to isolate the noise characteristics. (3) Determine the functional relationship between grayscale intensity and noise by selecting an appropriate fitting method. In this study, a 10th-order polynomial was chosen based on empirical observation, as it was simple and provided a good fit for our specific device’s non-linear noise characteristics. (4) Finally, determine the optimal values for spatial parameters $\sigma_{d}$ and β, through a traversal or search algorithm to match the observed spatial distribution.

**Figure 20:**
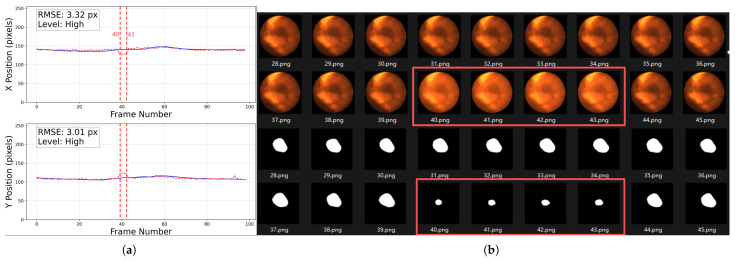
Abrupt variations in brightness and contrast. The left figure (**a**) shows a trajectory rated as *High*, the solid blue line represents the baseline trajectory, the dashed red line represents the predicted trajectory of the tumor center point. The tracking error markedly increases from frame 40 to 43 (as illustrated by the vertical dashed red lines). The right figure (**b**) presents the original CFIs and segmentation results, highlighting frames 40–43 (inside the red boxes) with strong brightness and contrast fluctuations. Note that the trajectory plot in (**a**) starts from frame 0, whereas the image filenames start from frame 1; therefore, frame 40 corresponds to position 39 in the trajectory plot.

**Figure 21:**
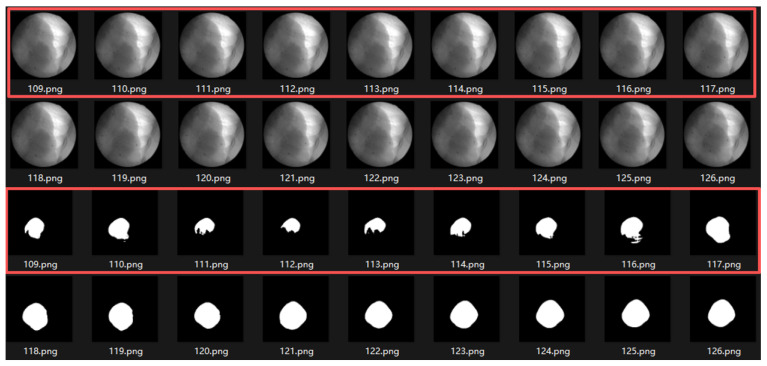
Motion-induced occlusion. During frames 109–117 (end of inhalation, inside the red boxes), the tumor enters the liver region and becomes partially occluded, resulting in blurred boundaries and decreased segmentation accuracy.

**Figure 22:**
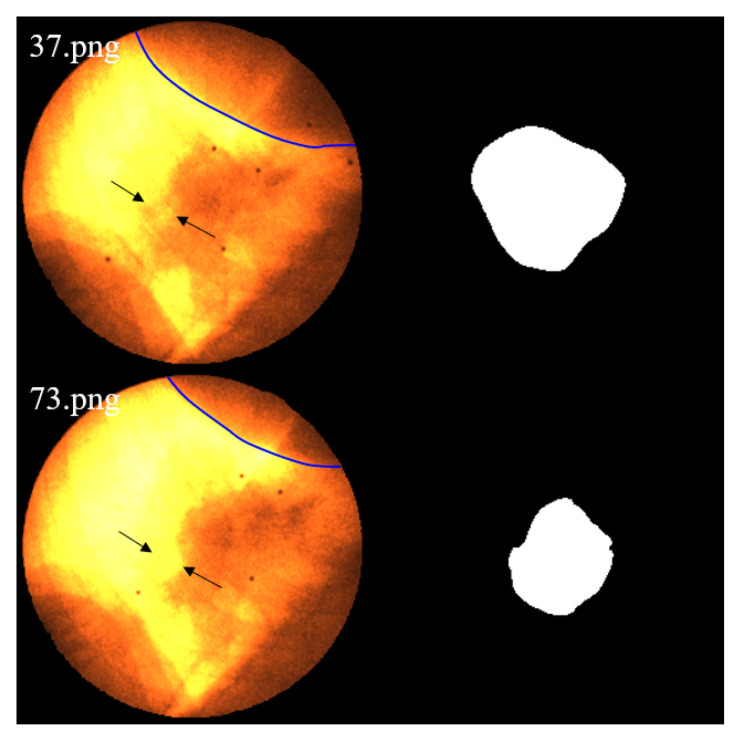
As lung expansion increases the proportion of lung regions in the image, the relatively low X-ray absorption of lung tissue leads to blurring of tumor-background boundaries and consequently degrades the segmentation performance. Image 37.png corresponds to the end-expiration phase, while 73.png shows the end-inspiration phase. The blue contour delineates the liver boundary. A marked reduction in liver area and a concurrent expansion of the pulmonary region are observed in 73.png. Additionally, the black arrows indicate regions where edges become blurred due to contrast variation.

In the analysis of frames with discrepancies between the tracking trajectory and the reference markers, we observed that these frames are typically accompanied by segmentation errors. The main contributing factors can be summarized as follows:

1. **Abrupt variations in brightness and contrast:** As shown in Figure 20, the left panel presents a trajectory rated as *High*, where the error significantly increases around frame 40. The right panel displays the original images and segmentation results from frames 28–45, where noticeable fluctuations in brightness and contrast occurred between frames 40–43, leading to reduced segmentation accuracy. Such variations are likely caused by the interference of MV treatment beams and scattered radiation.
2. **Tumor motion-induced occlusion and contrast fluctuation:**
  - When the tumor moves close to adjacent structures such as the liver, the boundary becomes severely blurred, resulting in decreased segmentation accuracy. As illustrated in Figure 21, the trajectory of this sequence is shown in Figure 14b. During frames 109–117, corresponding to the end of inhalation, the tumor enters the liver region and becomes partially occluded.
  - At the end of inhalation, the lung volume expands, and because lung tissue has weaker absorption of X-rays, the overall image contrast decreases. This effect is further amplified during grayscale conversion and style transfer, leading to degraded segmentation performance (Figure 22).
  Since both conditions often occur simultaneously at the end phases of inhalation, their combined influence further increases segmentation errors in some cases. In this study, six image sequences were affected by this effect, resulting in significant deviations in the maximum inspiratory stages, which in turn increased the predicted trajectory error to an E>5 pixels.
3. **Severe noise compounded with low contrast:** When the image suffers simultaneously from strong noise and insufficient contrast, the Restormer fails to restore sufficient detail, resulting in significantly degraded segmentation accuracy. As shown in Figure 14d, this sequence is affected not only by tumor motion but also by severe noise and low contrast, which exacerbate the overall error.

Our initial cohort included 10 patients; 3 of these patients were excluded from this study because our method is not suitable for tumors severely obscured by the liver, when the tumor is located in the lower lobe of the lung and obscured by tissues such as the liver, making its position difficult to discern even for the human eye. The accuracy of marker-less tracking based on tumor segmentation is insufficient for such cases. For these scenarios, alternative approaches such as regression-based motion detection using ROIs (e.g., as proposed by Hirai et al. [31]) or keypoint detection methods are recommended, as they do not rely on direct tumor contour segmentation. For tumor segmentation-driven approaches, enhancing tracking robustness and resolution in cases without severe obstructions, like the liver, could be a key pathway to improving clinical applicability.

The average processing time of our method is 179.8 ms per image, although it is smaller than the total system latency tolerance of 500 ms for real-time tumor tracking recommended by AAPM TG-76. However, it remains insufficient for the 30 fps requirement of the SyncTraX system. This is primarily due to the relatively high computational complexity of the Restormer and DUCK-Net models.

Although visually our method does reduce the style differences between X-ray and DRR images, in this study, image style transfer improved tumor tracking accuracy less than expected. To quantitatively analyze this trade-off, we compared the end-to-end performance of the workflow with and without the style transfer module. As summarized in Table 7, the results clearly show that while including style transfer improves tracking accuracy by approximately 5.6% in median Euclidean error, it increases the processing time by a substantial 76.6%.

This limited accuracy gain is primarily due to the inclusion of random noise and blur during DUCK-Net model training, which improves the model’s resilience to style differences. Our analysis indicates that for practical implementation, the substantial increase in processing time introduced by the style transfer step may outweigh its modest contribution to accuracy. Therefore, a streamlined workflow using DUCK-Net directly is a viable alternative. In future optimizations, adopting a more diverse data augmentation strategy for DUCK-Net could potentially further narrow the performance gap with the Restormer-based workflow, or even eliminate the need for a separate style transfer step. However, aggressive or poorly designed augmentations might introduce excessive stochasticity and noise, potentially causing the model to learn non-semantic features and thereby diminishing its ability to learn key anatomical features [39]. We therefore believe a targeted augmentation strategy could be more effective. For instance, introducing augmentations during training that are specifically designed to simulate the failure cases identified in our discussion—such as abrupt brightness changes from MV scatter, temporary tumor-liver occlusion, and low contrast at full inhalation—could be highly beneficial. This remains a critical and promising avenue for future optimization.

Furthermore, noise characteristics in fluoroscopic images were observed on two sets of equipment in this study, but their generalizability to other devices remains unclear. A clarification on the evaluation methodology is warranted. Our approach of aligning the mean positions of the predicted and ground-truth trajectories, while standard for comparing motion patterns, effectively cancels out systematic offsets. This means our reported accuracy primarily reflects the fidelity of tracking relative motion, rather than absolute spatial localization, and any constant positional bias would not be captured by this metric, which could be critical for beam gating. The scale of the dataset, which comprises nearly 30,000 images, renders manual tumor segmentation infeasible. Therefore, our study adopts the use of fiducial markers as a reference, which is a standard and practical methodology for validation in the marker-less tumor tracking field.

Future research could be expanded in several meaningful directions: 1. Incorporation of multi-center validation to assess robustness against variations in imaging protocols and equipment. This would enhance confidence in its broader clinical applicability. 2. Real-time optimization through model lightweighting or the use of more powerful GPUs to accelerate inference speed. 3. Multi-modal integration by incorporating signals from respiratory monitoring systems or other tumor tracking sources to improve robustness via multi-source information fusion, as well as clinical workflow integration that embeds the method into radiotherapy treatment planning systems and online image navigation platforms to develop a readily usable auxiliary localization tool.

## 5. Conclusions

In this study, we presented a novel framework for marker-less lung tumor tracking and established its feasibility using a cross-patient deep learning model. Our approach successfully tracked tumors with high accuracy across 100 radiation fields from 7 patients, achieving a median 3D Euclidean error of 1.53 mm (LR: 0.98±0.70 mm, SI: 1.09±0.74 mm, AP: 1.34±0.94 mm). The fiducial marker-based tracking system currently in clinical use at Yamaguchi University Hospital operates with an accepted localization accuracy of within 2 mm. Therefore, our achieved median 3D error of 1.53 mm is clinically promising. This result validates the potential of our method to overcome the need for patient-specific retraining. Furthermore, our analysis of segmentation failures provides critical insights into the remaining challenges, highlighting specific scenarios where future models need to be improved. A key finding from our analysis is the trade-off between accuracy and speed related to the style transfer step; for clinical scenarios demanding real-time performance, a streamlined workflow without style transfer is a viable option, sacrificing a minor degree of accuracy for a significant gain in processing speed.

This work serves as a comprehensive proof-of-concept, providing several foundational contributions. We designed a novel fluoroscopic image degradation model to more accurately simulate imaging physics. Furthermore, we introduced a segmentation strategy that leverages these simulated DRRs for training, circumventing the need for extensive annotations on clinical images. To our knowledge, this is the first study to demonstrate that millimeter-level tracking accuracy is achievable with a cross-patient model in this context. Finally, our end-to-end pipeline, which integrates temporal smoothing and a practical method for validating against fiducial markers, provides a complete blueprint for future development.

We acknowledge the primary limitation of this study is the small patient cohort. While the results are promising, the model’s performance on a broader, more diverse patient population remains to be confirmed. Therefore, the framework’s current validation should be considered a foundational but preliminary step toward a generalizable clinical solution.

Future work must prioritize extensive, multi-center validation to establish the robustness and true generalizability of our approach. Concurrently, performance optimization will be critical. Lightweighting the model through techniques like compression and hardware acceleration is essential to reduce the processing time from 180 ms per frame towards a clinically viable, real-time response. The ultimate goal is to evolve this technology into an integrated system for image-guided radiation therapy, enhancing the precision and safety of cancer treatment.

## Figures and Tables

**Table 1:** Computer Configuration.

Component	Specification
CPU	Intel Core i9-9940X
Memory	64 GB
GPU	GeForce RTX 3090
GPU Memory	24 GB GDDR6X

**Table 2:** Stochastic Strategy for Synthesizing Degraded Images.

Parameter	Random Strategy/Distribution	Physical Significance
$X_{c}$	$X_{c}∼N\left(0,30\right)$	Beam center offset
$\sigma_{d}$	$\sigma_{d}∼U\left(103,113\right)$	Central flatness radius
$B_{p},B_{m}$	$B_{p}∼U\left(0.35,0.37\right),B_{m}∼U\left(1.8,2.0\right)$	Base noise levels

**Table 3:** Training Configurations.

Parameter	Original (Restormer)	Ours
Number of GPUs	8	1
Number of Workers per GPU	8	4
Batch Size per GPU	8	4
Minibatch Sizes (Progressive)	[8, 5, 4, 2, 1, 1]	[4, 3, 2, 2, 1, 1]
Max Patch Size (GT Size)	384	320
Patch Sizes (Progressive)	[160, 192, 256, 320, 384]	[128, 160, 192, 256, 320]
Max Minibatch (Validation)	8	4

**Table 4:** Grading Standard Based on 2D Trajectory Error.

Grade	*E* (Pixels)	Description
Excellent	<3	Excellent performance in almost all frames.
High	3 ≤ *E* < 8	Few frames are not accurate enough.
Moderate	8 ≤ *E* < 13	Some frames had poor results.
Low	≥13	Poor accuracy in many frames.

**Table 5:** 2D trajectory evaluation comparison. All compared methods were evaluated on the exact same dataset.

Grade	*E*	Method 1 ^1^	Method 2 ^2^	Method 3 ^3^	Method 4 ^4^	Method 5 ^5^
Excellent	(0, 3]	22.73%	50%	48%	50%	51%
High	(3, 8]	39.9%	45%	47%	46%	45%
Moderate	(8, 13]	22.73%	4%	4%	3%	3%
Low	(13,∞]	14.64%	1%	1%	1%	1%

^1^ Existing method [32]. ^2^ Omitting style conversion of the proposed method. ^3^ Restormer trained with the baseline dataset of the proposed method. ^4^ Proposed method without LPF. ^5^ Proposed method.

**Table 6:** 3D trajectory evaluation comparison.

	Method 1 ^1^	Method 2 ^2^	Method 3 ^3^
LR	0.98 ± 0.67 mm	1.00 ± 0.69 mm	0.98 ± 0.70 mm
SI	1.14 ± 0.76 mm	1.15 ± 0.77 mm	1.09 ± 0.74 mm
AP	1.34 ± 0.93 mm	1.35 ± 0.91 mm	1.34 ± 0.94 mm
MEE*	1.62 mm	1.64 mm	1.53 mm
IQR*	1.04 mm	0.99 mm	1.04 mm

MEE*: Median euclidean error. IQR*: Interquartile Range. ^1^ Omitting style conversion of the proposed method. ^2^ Restormer trained with the baseline dataset of the proposed method. ^3^ Proposed method.

**Table 7:** Performance trade-off analysis of the style transfer module.

Workflow Configuration	MEE (mm)	Processing Time (ms/Frame)
Without Style Transfer	1.62	101.8
With Style Transfer (Proposed)	1.53	179.8
Change	5.6% performance improvement	Time increased by 76.6%

## Data Availability

The images are not available, but for more information including scripts, trained models, etc., see https://github.com/YongxuanYan/General_Markerless_Tumor_Tracking accessed on 7 September 2025.

## Abbreviations

The following abbreviations are used in this manuscript:

DRR	Digitally Reconstructed Radiograph
CFI	Color Fluoroscopic Image
IGRT	Image-Guided Radiation Therapy
OAR	Organ at Risk
ROI	Region of Interest
DNN	Deep Neural Network
RMSE	Root Mean Square Error
LR	Left–Right (direction)
SI	Superior–Inferior (direction)
AP	Anterior–Posterior (direction)
SBRT	Stereotactic Body Radiation Therapy
HU	Hounsfield Unit
fps	Frames Per Second

## References

[1] Ferlay J., Ervik M., Lam F., Laversanne M., Colombet M., Mery L., Piñeros M., Znaor A., Soerjomataram I., Bray F. (2024). Global Cancer Observatory: Cancer Today. https://gco.iarc.who.int/today.

[2] Wang L., Lynch C., Pitroda S.P., Piffkó A., Yang K., Huser A.K., Liang H.L., Weichselbaum R.R. (2024). Radiotherapy and immunology. J. Exp. Med..

[3] Dona Lemus O.M., Cao M., Cai B., Cummings M., Zheng D. (2024). Adaptive radiotherapy: Next-generation radiotherapy. Cancers.

[4] Zhu H., Chua M.L.K., Chitapanarux I., Kaidar-Person O., Mwaba C., Alghamdi M., Mignola A.R., Amrogowicz N., Yazici G., Bourhaleb Z. (2024). Global radiotherapy demands and corresponding radiotherapy-professional workforce requirements in 2022 and predicted to 2050: A population-based study. Lancet Glob. Health.

[5] Bertholet J., Knopf A., Eiben B., McClelland J., Grimwood A., Harris E., Menten M., Poulsen P., Nguyen D.T., Keall P. (2019). Real-time intrafraction motion monitoring in external beam radiotherapy. Phys. Med. Biol..

[6] Wang Y., Lombardo E., Thummerer A., Blöcker T., Fan Y., Zhao Y., Papadopoulou C.I., Hurkmans C., Tijssen R.H., Görts P.A. (2025). TrackRAD2025 challenge dataset: Real-time tumor tracking for MRI-guided radiotherapy. Med. Phys..

[7] Zhou D., Nakamura M., Mukumoto N., Matsuo Y., Mizowaki T. (2023). Feasibility study of deep learning-based markerless real-time lung tumor tracking with orthogonal X-ray projection images. J. Appl. Clin. Med. Phys..

[8] Li G. (2022). Advances and potential of optical surface imaging in radiotherapy. Phys. Med. Biol..

[9] De Crevoisier R., Lafond C., Mervoyer A., Hulot C., Jaksic N., Bessières I., Delpon G. (2022). Image-guided radiotherapy. Cancer/Radiothérapie.

[10] Rammohan N., Randall J.W., Yadav P. (2022). History of technological advancements towards MR-Linac: The future of image-guided radiotherapy. J. Clin. Med..

[11] Chrystall D., Mylonas A., Hewson E., Martin J., Keall P., Booth J., Nguyen D.T. (2023). Deep learning enables MV-based real-time image guided radiation therapy for prostate cancer patients. Phys. Med. Biol..

[12] Lu Q., Guo D., Wei Y., Zheng J., Li J. (2023). Role of gastrointestinal ultrasound in image-guided radiation therapy: A review. J. Radiat. Res. Appl. Sci..

[13] Liu X., Geng L.S., Huang D., Cai J., Yang R. (2024). Deep learning-based target tracking with X-ray images for radiotherapy: A narrative review. Quant. Imaging Med. Surg..

[14] Campbell W.G., Miften M., Jones B.L. (2017). Automated target tracking in kilovoltage images using dynamic templates of fiducial marker clusters. Med. Phys..

[15] Li R., Mok E., Chang D.T., Daly M., Loo Jr B.W., Diehn M., Le Q.T., Koong A., Xing L. (2012). Intrafraction verification of gated RapidArc by using beam-level kilovoltage X-ray images. Int. J. Radiat. Oncol. Biol. Phys..

[16] Van der Horst A., Wognum S., Fajardo R.D., De Jong R., Van Hooft J.E., Fockens P., Van Tienhoven G., Bel A. (2013). Interfractional position variation of pancreatic tumors quantified using intratumoral fiducial markers and daily cone beam computed tomography. Int. J. Radiat. Oncol. Biol. Phys..

[17] Casutt A., Kinj R., Ozsahin E.M., von Garnier C., Lovis A. (2022). Fiducial markers for stereotactic lung radiation therapy: Review of the transthoracic, endovascular and endobronchial approaches. Eur. Respir. Rev..

[18] Oudin V., Salleron J., Marchesi V., Peiffert D., Khadige M., Faivre J.C. (2023). CyberKnife^®^ stereotactic radiation therapy for stage I lung cancer and pulmonary oligometastases: Is fiducial implantation still relevant?—A cohort study. J. Thorac. Dis..

[19] Casutt A., Noirez L., Bernasconi M., Koutsokera A., Beigelman-Aubry C., Kinj R., Ozsahin E.M., Durham A.D., von Garnier C., Lovis A. (2021). Endobronchial coil spring fiducial markers for CyberKnife^®^ stereotactic body radiation therapy. Respirology.

[20] Khullar K., Dhawan S.T., Nosher J., Jabbour S.K. (2021). Fiducial marker migration following computed tomography-guided placement in the liver: A case report. AME Case Rep..

[21] Mylonas A., Booth J., Nguyen D.T. (2021). A review of artificial intelligence applications for motion tracking in radiotherapy. J. Med. Imaging Radiat. Oncol..

[22] Ronneberger O., Fischer P., Brox T. (2015). U-net: Convolutional networks for biomedical image segmentation. Proceedings of the International Conference on Medical Image Computing and Computer-Assisted Intervention.

[23] Vaswani A., Shazeer N., Parmar N., Uszkoreit J., Jones L., Gomez A.N., Kaiser Ł., Polosukhin I. (2017). Attention is all you need. Adv. Neural Inf. Process. Syst..

[24] He K., Zhang X., Ren S., Sun J. Deep residual learning for image recognition. Proceedings of the IEEE Conference on Computer Vision and Pattern Recognition.

[25] Goodfellow I.J., Pouget-Abadie J., Mirza M., Xu B., Warde-Farley D., Ozair S., Courville A., Bengio Y. (2014). Generative adversarial nets. Adv. Neural Inf. Process. Syst..

[26] Roggen T., Bobic M., Givehchi N., Scheib S.G. (2020). Deep Learning model for markerless tracking in spinal SBRT. Phys. Medica.

[27] He X., Cai W., Li F., Fan Q., Zhang P., Cuaron J.J., Cerviño L.I., Li X., Li T. (2021). Decompose kV projection using neural network for improved motion tracking in paraspinal SBRT. Med. Phys..

[28] Terunuma T., Sakae T., Hu Y., Takei H., Moriya S., Okumura T., Sakurai H. (2023). Explainability and controllability of patient-specific deep learning with attention-based augmentation for markerless image-guided radiotherapy. Med. Phys..

[29] Wu Y., Kirillov A., Massa F., Lo W.Y., Girshick R. (2019). Girshick R. Detectron2. https://github.com/facebookresearch/detectron2.

[30] Lin T.Y., Maire M., Belongie S., Hays J., Perona P., Ramanan D., Dollár P., Zitnick C.L. (2014). Microsoft coco: Common objects in context. Proceedings of the European Conference on Computer Vision.

[31] Hirai R., Sakata Y., Tanizawa A., Mori S. (2019). Real-time tumor tracking using fluoroscopic imaging with deep neural network analysis. Phys. Medica.

[32] Yan Y., Fujii F., Shiinoki T., Liu S. (2024). Markerless Lung Tumor Localization From Intraoperative Stereo Color Fluoroscopic Images for Radiotherapy. IEEE Access.

[33] Nafchi H.Z., Shahkolaei A., Hedjam R., Cheriet M. (2017). CorrC2G: Color to gray conversion by correlation. IEEE Signal Process. Lett..

[34] Shiinoki T., Fujii F., Fujimoto K., Yuasa Y., Sera T. (2020). A novel dynamic robotic moving phantom system for patient-specific quality assurance in real-time tumor-tracking radiotherapy. J. Appl. Clin. Med. Phys..

[35] Sarno A., Andreozzi E., De Caro D., Di Meo G., Strollo A., Cesarelli M., Bifulco P. (2019). Real-time algorithm for Poissonian noise reduction in low-dose fluoroscopy: Performance evaluation. Biomed. Eng. Online.

[36] Huda W., Abrahams R.B. (2015). Radiographic techniques, contrast, and noise in x-ray imaging. Am. J. Roentgenol..

[37] Zamir S.W., Arora A., Khan S., Hayat M., Khan F.S., Yang M.H. Restormer: Efficient transformer for high-resolution image restoration. Proceedings of the IEEE/CVF Conference on Computer Vision and Pattern Recognition.

[38] Dumitru R.G., Peteleaza D., Craciun C. (2023). Using DUCK-Net for polyp image segmentation. Sci. Rep..

[39] Koohpayegani S.A., Tejankar A., Pirsiavash H. Mean shift for self-supervised learning. Proceedings of the IEEE/CVF International Conference on Computer Vision.

